# Stress-Shot-Peened Leaf Springs Material Analysis through Nano- and Micro-Indentations

**DOI:** 10.3390/ma14174795

**Published:** 2021-08-24

**Authors:** Maria Pappa, Georgios Savaidis, Nikolaos Michailidis

**Affiliations:** 1 Physical Metallurgy Laboratory, School of Mechanical Engineering, Aristotle University of Thessaloniki, GR-54124 Thessaloniki, Greece; nmichail@auth.gr; 2 Laboratory of Machine Elements and Machine Design, School of Mechanical Engineering, Aristotle University of Thessaloniki, GR-54124 Thessaloniki, Greece; gsavaidis@auth.gr

**Keywords:** nanoindentation, microstructural analysis, mechanical properties, shot peening

## Abstract

Heat-treated and shot-peened lightweight steels with demanding requirements for durability are applied in high-performance automotive leaf springs. Due to their heat-treatment they exhibit degraded properties in the surface-near area compared to the core. This area, which may extend until 300 μm from the surface to the core, experiences the highest bending stresses at operation. The microstructure in the surface and sub-surface layers determines the mechanical performance as well as the wear resistance. The present study refers to the material properties of a stress shot-peened 51CrV4 steel at various depths from the surface. The effect of the manufacturing process has been captured both by Vickers micro-hardness measurements and nanoindentation. The latter combined with a Fine Element Method (FEM)-based algorithm enables the determination of variations in the material’s stress–strain curves over the affected layers, which translate to internal stress changes. The nanoindentation technique has been applied here successfully for the first time ever on leaf springs. The combination of microstructural analysis, microhardness and nanoindentation captures the changes of the treated material, offering insights on the material characteristics, and yielding accurate elastoplastic material properties for local, layered-based analysis of the components’ mechanical performance at operational loading scenarios, i.e., in the framework of stress shot-peening simulation models.

## 1. Introduction

High strength steels with ultimate tensile strength >1600 MPa after quenching and tempering are used in technological areas, where the superior strength and enhanced fatigue life are essential for the overall safety of the structure. Most demanding applications exploiting the full potential of such steels is in load-bearing automotive axle suspension components, of the respective industry being at the cutting edge of research and development. The actual status of relevant surface treating processes, in particular the stress shot-peening (SSP) for Z-links, antiroll bars, leaf or coil springs and torsion bars can be described by the methods illustrated in [1,2,3,4], although these articles are ten to twenty years old. Nowadays, the SSP process is considered the most promising and effective way to enhance the fatigue properties of suspension components on an industrial scale. It has been adopted by all major manufacturers as a time- and cost-effective treatment for at least doubling the fatigue life of their products, especially leaf springs [1]. However, the SSP process control and optimization are very challenging tasks of high complexity: shot material, geometry and hardness, shot velocity and incidence angle, degree of coverage and tensile prestress interact with the already uncertain state of the area of application (decarburized surface with reduced strength, inevitably induced by the preceding heat treatment). As a result, the surface roughness, work hardening, and residual stresses are significantly affected. Over the past two decades research activities were focused on the SSP simulations to elaborate optimal and reproducible processes. Unfortunately, this knowledge has so far failed to be successfully transferred to suspension components, mainly because the material properties acting at the failure-critical decarburized surface (a) are very difficult to be obtained accurately at a local level and (b) often differ significantly from those of the core [5]. Due to these handicaps and the multi-parametric and very complex technical nature of the involved manufacturing procedures, the process optimization is still a labor-intensive task that relies on individual experience and requires many experimentation loops. The present work aims to overcome those deficiencies by determining the material characteristics of industrially treated SSP leaf springs after heat treatment and tapering (HTT) on the surface and the subsurface, employing micro- and nanoindentation combined with sophisticated FEM-based modeling techniques of the nanoindentation. The particular innovation of the current work lies in the novel application of nanoindentation on leaf springs to characterize the critical surface and subsurface properties of the treated material and determine “layered” elastoplastic stress-strain curves, that can feed capable SSP simulation models to increase their calculation efficiency, contributing in the design and the more realistic modeling of the processed materials towards product optimization. The results presented here have been extracted from investigations performed within the framework of the LIGHTTECH project (LIGHTTECH—Innovative Approaches of Stress Shot Peening and Fatigue Assessment for the Development of Lightweight, Durability-Enhanced Automotive Steel Leaf Springs) [6].

## 2. Materials and Methods 

The material used for high-performance leaf springs and studied here is 51CrV4 (1.8159) steel grade. Its chemical composition according to DIN EN 10089 (Hot rolled steels for quenched and tempered springs—Technical delivery conditions) is shown in Table 1. The aluminum content is only 0.001 wt.% (information that is not specified in DIN EN 10089) due to the silicon deoxidation.

**Figure 1:**
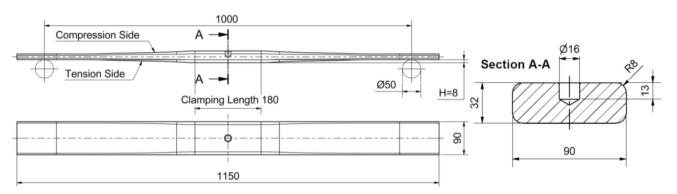
Technical drawing of the parabolic leaf spring specimens—dimension in [mm].

HTT is a process that leads to the hardening of steel. This is largely due to the presence of C atoms in the interference sites in the body-centered tetragonal (BCT) crystalline martensite. Carbon occupies the same interfacial positions in austenite and martensite. However, while austenite causes symmetric deformation, martensite causes non-symmetric deformation, which is the predominant cause for the solid solution to solidify. The industrial HTT treatment performed on the real size leaf springs involved: heating of the raw flat bar material at 850 °C, morphing of the leaf spring geometry via rollers, quenching in oil of approximately 60 °C, and tempering at 450 °C. Figure 1 contains the technical drawing of the leaf springs. The width is constant to 90 mm along the length of the specimen. The central (or clamped) area of the leaf has a constant thickness of 32 mm over a length of 180 mm. The arms on the left and right sides have a parabolically decreasing thickness along their length, as the distance from the central area is increasing. Table 2 contains characteristic values of the thickness distribution of the leaf. Notice that the leaves are subjected to three-point bending during operation, where a vertical load is introduced in the central area and the support of the leaves occurs is facilitated by two cylinders with diameter of 50 mm positioned at the two ends over a span of 1000 mm. The parabolic arms have been designed with the purpose of experiencing approximately constant stresses over their whole length during the three-point bending operation.

The surface properties are experimentally determined using small specimens extracted from the parabolic arms of the leaf springs, after HTT and SSP processes, sequentially. The specimens taken after the HTT process are thoroughly inspected to acquire all necessary information regarding the microstructure and decarburization [7] state of the surface that will undergo the SSP treatment at a next stage. This way, the underlying material properties can be described in detail and correlated against the stages after the SSP treatment. Further investigations including all scales of hardness and indentation (in nano- and micro-scale) are also performed using the same specimens. 

The microstructural inspection resides on specimens specifically prepared for this purpose. The preparation of such is a meticulous and time-consuming process that consists of several sequential stages that ensure the repeatability and robustness of the results, while eliminate those parameters that could tamper with the outcome due to mishandling or human error. The first step is to cut out a relatively large portion out of the leaf specimen (>50 mm in length) using a medium-scale fixed cutting wheel with constant water cooling of the cutting area. This ensures a temperature level below 100 °C during the whole cutting process and hinders metallographic changes or transformations that would confuse the end-result of the investigation. The next stage involves the extraction of smaller specimens using a Buehler IsoMet low-speed sectioning diamond wheel (Lake Bluff, IL, USA) [8] that removes most of the material of the previous cuts, to further eliminate even the slightest suspicion of ending up with unwittingly thermally and mechanically influenced parts due to cutting conditions. The remaining material is then mounted on a Bakelite housing (a thermally conductive phenolic resin usually used for such cases) to facilitate the proper specimen orientation, marking and labeling as well as the convenient handling during preparation and inspection. The mounted samples were then mechanically ground in several sequential steps employing BUEHLER Vanguard^TM^ 2000 (Lake Bluff, IL, USA) automatic sample preparation system [9]. Chemical etching with NITAL 5% was performed to reveal the microstructure. Following etching, the samples were washed in alcohol and rapidly dried by the flow of warm air.

To quantify the decarburization intensity and extent and correlate the anticipated strength reduction with a practical mechanical property such as the tensile strength, several series of micro-hardness measurements were conducted at all areas of interest. Hardness is more of a characteristic mechanical property, rather than a fundamental physical property of a material. It is defined as the resistance to indentation and is determined by measuring the dimensions of the permanent impression caused by an indenter of a specific geometry at a predefined load. The Vickers micro-hardness was selected and profiles over the depth from the surface were allocated, verifying the decarburization zone observed through microscopy and translating its characteristics to tangible quantitative values. A Shimadzu type M (Schimadzu, Kyoto, Japan) microhardness tester was applied to carry out all Vickers microhardness measurements.

To analyze the microstructure and the size of Vickers impressions performed on the specimens, optical microscopy by a LEICA DM4000 (Wetzlar, Germany) [10] and scanning electron microscopy (SEM) by Thermo Scientific Phenom ProX (Waltham, MA, USA) [11] were applied.

Nanoindentation is used for testing the mechanical properties of materials, facilitated by high-precision instrumentation in the nanometer scale [12]. The method is applied to register the course of the applied load versus the resulting indentation depth (displacement). It consists of two steps, the so-called loading stage and the unloading one. During the loading stage, an indentation load applied on the indenter, forces it to penetrate into the specimen. The load is gradually applied and at the same time the indentation depth is measured. After the load is gradually removed (unloading stage), due to the resulting material plastic deformation, there is a remaining depth [12]. In 2008, the ISO/TR 29381 was published [13], allowing for the evaluation of tensile properties of metallic materials by instrumented indentation. The evolution of FEM-based algorithms in the evaluation of nanoindentation results offers advanced capabilities in determining the exact contact between the indenter and the test piece, thus enabling the accurate calculation of the material hardness and stress–strain curves [14,15]. The loading stage is the one that is used to translate the force versus indentation depth curves into stress–strain mechanical behavior, while the unloading stage and the remaining indentation depth can be used to verify the measurement and translate the procedure to nano-hardness values.

A nanoindentation device was developed from the research members to address the specific needs and meet the set requirements. The instrument has a motorized workbench with a working range of 50 x 140 mm and a positioning accuracy on each of the planar axes of 1 μm. The load may range from 0.1 to 800 mN with a resolution of 0.05 mN, while the indentation depth may reach the value of 30 μm, with a resolution of 0.06 nm. In order to achieve such a high resolution, the load application is guided by a dual-stage encoder, i.e., a conventional mechanical macro-stage and a second piezoelectric stage of very high accuracy. Berkovich, Vickers, Knoop pyramid-like and 0.5–2 mm ball diamond indenters can be employed to acquire the respective force versus indentation depth curves. All functions of the indentation procedure are fully automated, and the apparatus is able to record a large number of indentations over a small amount of time. 

## 3. Results and Discussion

### 3.1. Microstructural Analysis

**Figure 2:**
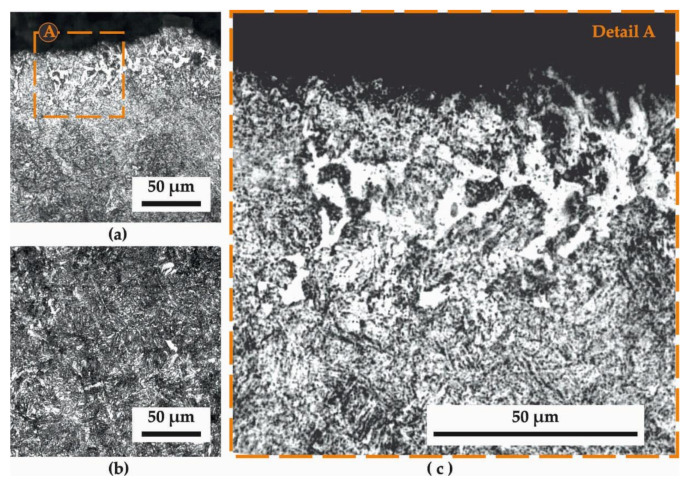
Typical microstructure of the heat treatment and tapering (HTT) specimens (longitudinal section): (**a**) close to the surface; (**b**) at the core (**c**) close to the surface at higher magnification.

**Figure 3:**
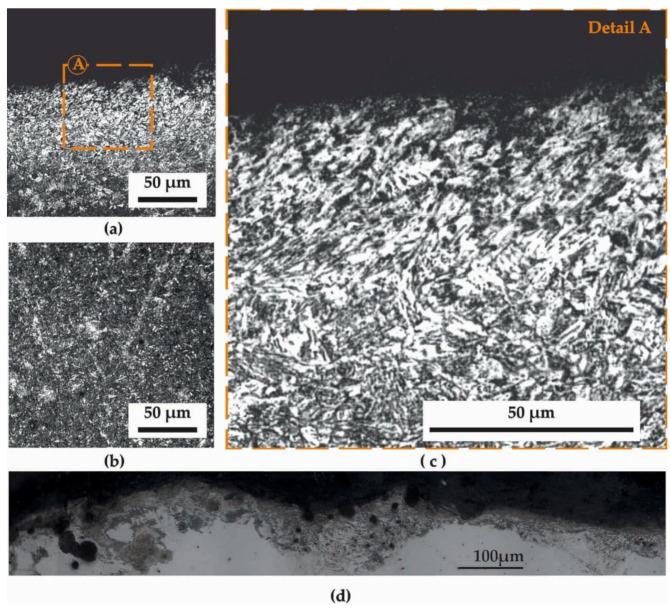
Typical microstructure of the HTT + stress shot-peening (SSP) specimens (longitudinal section): (**a**) close to the surface; (**b**) at the core (**c**) close to the surface at higher magnification and (**d**) surface without chemical etching revealing the distortion caused by SSP.

Figure 2 presents the microstructure in a longitudinal section of the leaf spring surface and subsurface after HTT, and Figure 3 the corresponding section after HTT+SSP treatments. Magnifications of (a) 500× (images at the left-hand side) and (c) 1000× (images at the right-hand side), while for reasons of comparison and referencing, the (b) microstructure of the core of the specimens is presented at the bottom-left of the figures. This microstructure is according to those of the heat-treated core surfaces that have been studied in [16], where nanoindentations and stress strains were discussed for quenching and tempering through a laser-based surface modification process of a dual-phase spring steel.

The microstructure of Figure 2a is a typical tempered-martensite one. For steels with up to 0.6% carbon content (51CrV4 has a carbon content in the range of 0.47–0.55%) lath martensite is expected to form, as characteristically depicted in Figure 2c. Besides the martensitic microstructure, retained austenite is also present as the result of tempering. The tempering process allows nucleation of cementite by gradual stress relieving, until the previous overabundance of martensite reaches the required equilibrium between martensite and retained austenite. This is done to balance the competing behavior between strength/hardness and brittleness (i.e., high content of martensite makes steel brittle, while low content makes it weak/soft). It is evident that there is a certain extent of partial decarburization up to about a characteristic depth ranging between 100 and 300 μm with a decreasing intensity towards the core (“washed-out” white areas depict the lack of carbon). The decarburization depth and qualitative intensity can be estimated according to the standards [17,18], while the reasons for its occurrence should be sought among the surface treatments applied during the leaf production, i.e., the heat treatment and the hot tapering, as both are high-temperature processes that may promote the reaction of carbon with oxygen towards the formation of CO_2_. 

The observation of the microstructure after SSP (Figure 3a) post-HTT, reveals the acupuncture martensite in the structure of the material, which is markedly different from the other two cases (core and HTT). What has changed in this case is the orientation of the needle-like structure of the martensite close to the surface, which has been severely distorted by the high-velocity impact of the shots during SSP. In particular, the uppermost surface layer seems to follow “swirling” patterns around locations where the impacts released their bursts of kinetic energy. In an effort to achieve a quick and clear quantification of the SSP zone, it was proven very efficient to capture by optical microscopy the microstructure of the longitudinal section just after polishing without any chemical etching, as Figure 3d presents.

**Figure 4:**
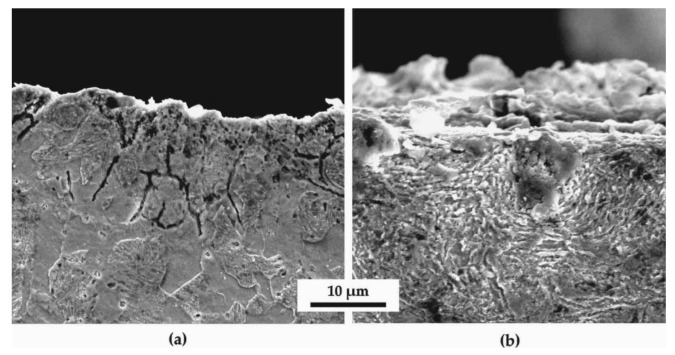
Scanning electron microscope (SEM) images of longitudinal sections close to the surface of the (**a**) HTT and (**b**) HTT+SSP at a magnification of 6000×.

Figure 4 shows various SEM images at higher magnifications (6000x) than optical microscopy allows. Zooming on Figure 4b of the SSP treatment after HTT, it can be noticed that there is a radial-shaped pattern, illustrating the concentration of flows around the impact site of a shot. On the other hand, the HTT specimen does not seem to present such microstructural distortion, as shown in Figure 4a.

**Figure 5:**
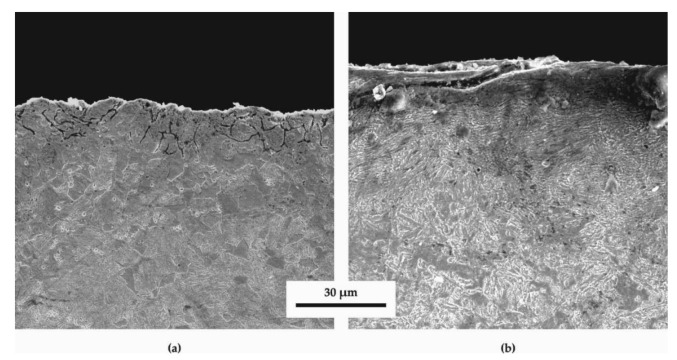
SEM images of longitudinal sections close to the surface of the (**a**) HTT and (**b**) HTT + SSP specimens.

Figure 5 shows SEM images at lower magnifications (2500x) for the cases of (a) HTT and (b) HTT+SSP specimens, which offer a broader view of the previously mentioned microstructural deformation of HTT+SSP (Figure 5b), with the orientations of the lath martensite being shifted to directions other than those evidenced in the core, and in comparison, to the completely different microstructure of the HTT specimen (Figure 5a), as well.

### 3.2. Micro-Hardness Analysis

**Figure 6:**
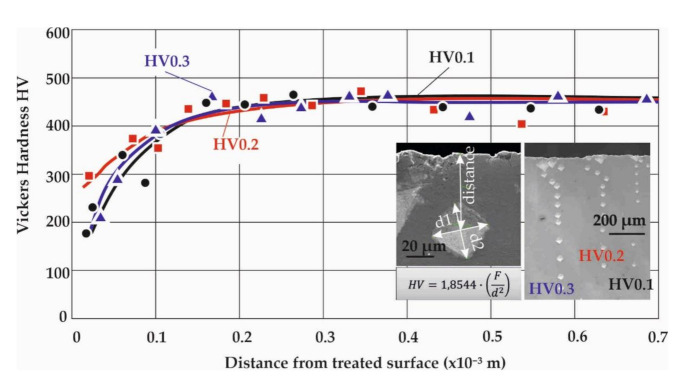
Comparison of Vickers hardness distribution over the distance from the surface, measured after applying 0.1, 0.2 and 0.3 kgf (indentation time 15 sec); exemplary study on an HTT specimen.

The Vickers micro-hardness measured on a sample might depend on the selected set of testing parameters alone from the material properties. Here, a major affecting parameter is the weight applied on top of the indenter. This should be carefully selected to allow for the acquaintance of results that will be realistic and practical for further analysis. Therefore, a sensitivity analysis was initially performed to determine the right weight to use. Figure 6 exemplarily illustrates the differences captured when measuring at the same area of a specimen using 0.1, 0.2 and 0.3 kilogram-force (kgf), for a duration of 15 sec. 

The Vickers microhardness is determined by measuring the two diagonals of the rectangular impression through optical and electron microscopy. To achieve a high resolution of the measured hardness distribution over the distance from the surface, a small interval of about 20–25 μm was selected between the impressions and multiple measurements were performed in an inclined pattern, especially near the surface edge, to avoid interference between successive impressions. In all the examined cases, the different applied weight force did not yield to significant changes in the measured Vickers hardness, with the lower loads producing larger scatter-bands, since they capture only the localized hardness, especially in regions where multiple phases coexist, while the impressions are not as sharp and clear as those of a 0.3 kgf load.

It is evident that the “swirled” (HTT+SSP) and decarburized (HTT) microstructure creates a relatively wider scatter of measurements for the 0.1 kgf case due to the higher sensitivity of the method at severely distorted discrete areas. Hence, 0.3 kgf is selected to better characterize the material state, particularly for the decarburized zone.

**Figure 7:**
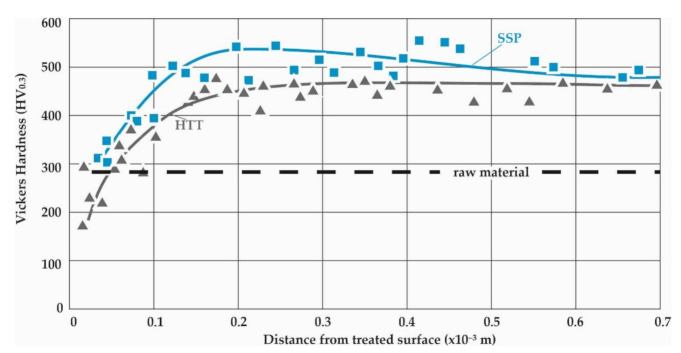
Vickers micro-hardness measurements, average distributions and scattering of results on the HTT and HTT+SSP specimens along the distance from the surface.

Figure 7 presents the Vickers micro-hardness measurements conducted on the longitudinal sections close to the surface of the HTT and HTT+SSP specimens. To increase the accuracy of the results, the dimensions of the impressions and their distance from the surface were measured employing SEM microscopy.

It is noticeable that all measurements converge to almost the same distribution towards the surface, regardless of the SSP treatment, which clearly dictates that the decarburization during HTT is the dominant cause of the strength reduction towards the surface. SSP after HTT increases the micro-hardness close to the surface, but the effect of decarburization is still evident. Considering that the tensile strength of the raw material has been measured to approximately 1000 N/mm^2^ [19], which corresponds to a HV of about 300, it can be easily deducted that there is a decarburization layer of nearly 60 μm which fades out at 100 μm to 200 μm. Both HTT and HTT+SSP Vickers microhardness distributions converge at their core (distance from the surface >0.6 mm) to almost the same microhardness value, as both the decarburization (by HTT) and internal (residual) stresses (by SSP) are surface phenomena that are eliminated at the core of the specimens.

### 3.3. Nanoindentation and Layered σ-ε Properties

**Figure 8:**
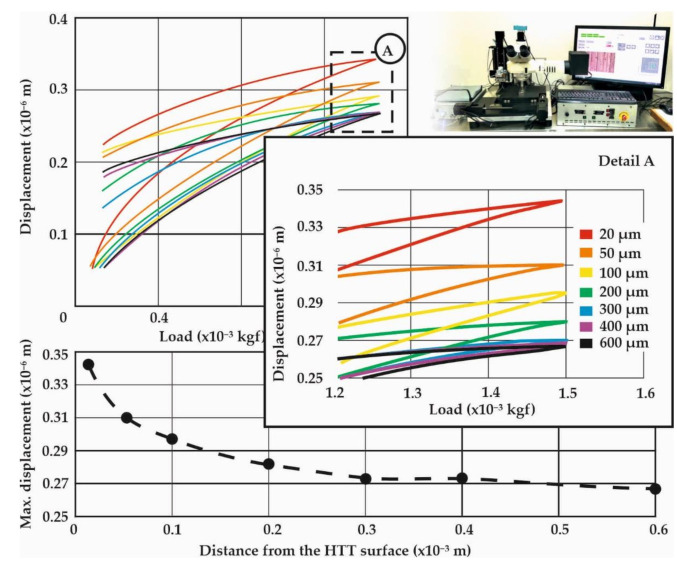
Nanoindentation load-displacement diagrams for the HTT leaf spring specimens at characteristic distances from the surface.

**Figure 9:**
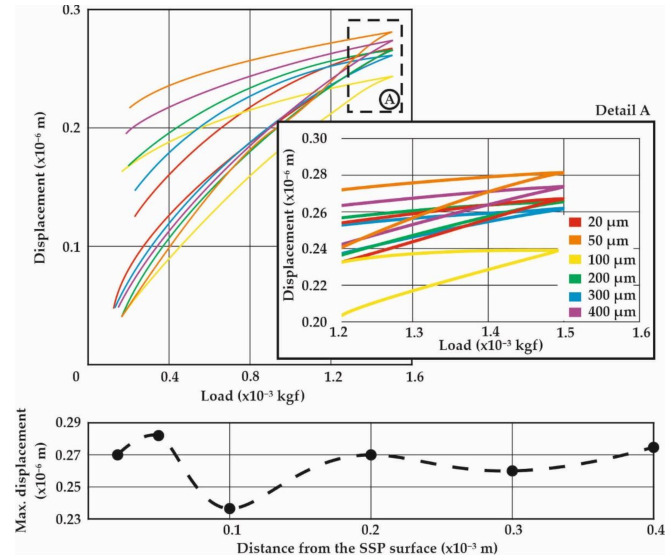
Nanoindentation load-displacement diagrams for the HTT+SSP leaf spring specimens at characteristic distances from the surface.

The aim of the nanoindentation investigations was to determine first the layered properties of the HTT leaf specimens, and to ultimately gain detailed insights on the surface and sub-surface mechanical properties after the SSP process. Therefore, several measurements were conducted on different sites and subsurface distances (at least 30 measurements on each distance), that were statistically evaluated to present the average, as well as the scatter of each set [20]. The maximum applied load in all the investigated cases was kept constant at 15 mN (1.5 × 10^−3^ kgf) which results in indentation depths up to 0.3 μm. Figure 8 presents the nanoindentation load-displacement diagrams of the HTT leaf specimens for the characteristic distances from the surface of 20, 50, 100, 400 and 600 μm, while Figure 9 represents the corresponding HTT+SSP ones. Because of the decarburization of HTT specimens, a gradual strengthening is expected towards the core up to 600 μm. It is clearly indicated by the nanoindentation measurements that the site after 300 μm can be considered as the core material with zero decarburization.

The gradual strengthening of the HTT material towards the core is evident through the decrease of the indentation displacement, as the distance from the surface increases. The 20 μm site corresponds to the maximum displacement, representing the decarburized surface layer, while after 300 μm it is constant and stiff. 

The diagrams at the bottom of Figure 8 and Figure 9 present the course of maximum indentation depth versus the distance from the treated surface, which correspond well to the Vickers hardness relations. The SSP procedure resulted in an enhanced behavior, especially at a distance from the surface of 100 μm, where both the micro- and nano- indentations revealed a hardening behavior. This can be attributed to the maximum plastic deformation and residual stresses caused by the shot peening process appearing at this distance from the surface.

**Figure 10:**
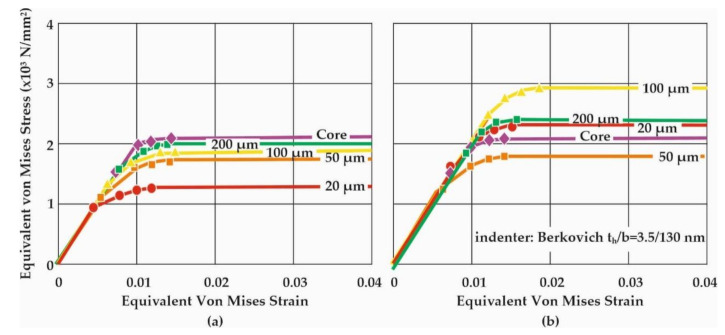
Equivalent von Mises stress–strain plots characterizing the behavior of (**a**) HTT and (**b**) HTT+SSP leaf spring specimens at distinct distances from the surface, calculated for the compressive nature of nanoindentation.

With the aid of the maximum penetration depth, the remaining depth, and a number of further parameters related to the nanoindentation procedure, the elasticity modulus can be approximately determined through analytical evaluation methods [21,22]. The ‘‘SSCUBONI’’ algorithm presented in [22], simulating in a stepwise manner the nanoindentation, enables the extraction of materials’ stress–strain laws [23,24]. Figure 10 presents the stress–strain curves (σ-ε) of the corresponding load-displacement measurements for HTT and HTT+SSP, respectively, as already displayed in Figure 8 and Figure 9.

The calculated stress–strain behavior is in line both qualitatively with the reasoning of decarburization (higher strength towards the core) and quantitatively with the micro-hardness measurements conducted at the same locations. This means that the nanoindentation method can be successfully utilized to acquire a very detailed representation of the layered mechanical behavior in terms of σ-ε curves rather than plain hardness values which can be correlated solely with a tensile strength value. These σ-ε curves are essential for the realistic modelling of the SSP process, since they serve as the foundations of the analysis, whose legitimacy and accuracy will dominantly determine the final outcome.

This work highlights the novel quantitative determination of the stress–strain curves of surface and subsurface material after exposure to SSP processes of leaf springs after heat treatment. This knowledge is catalytic towards understanding the material behavior and will enable the development of realistic computational models to assess failure. The diagrams of Figure 6, Figure 7, Figure 8 and Figure 9 showcase the gradient of plastic deformation which can be quantified and translated to stress and strain as presented in Figure 10. The intense plastic deformation of the subsurface is also evidenced by the microstructure of the longitudinal sections [25,26].

## 4. Conclusions

Surface and sub-surface properties of the HTT and SSP processes on leaf specimens have been thoroughly studied, both qualitatively and quantitatively. The microstructures along the profile thickness have been identified including the decarburization zone intensity and extent caused by the heat treatment, employing a series of micro-hardness measurements along the distance from the surface. Additionally, the SSP-affected zone was determined on the heat-treated leaf springs through microstructural analysis and indentations at various scales. In particular, the Vickers micro-hardness measurements revealed significant decrease of the material hardness in the decarburized surface layers compared to that of the core. The nanoindentations performed along the distance from the treated surface down to the core proved the work-hardening effect on the surface layers as an outcome of the severe deformation imposed by the SSP process. The SSP process may cause severe plastic deformation on the surface and subsurface of the specimens. The microstructural changes are manifested through a “swirled-like” type of grains as documented by SEM micrographs. However, this type of grain was extremely difficult to characterize in terms of grain size due to the high degree of distortion. Further analysis of the nanoindentation load-displacement curves by FEM-based algorithms showed significant enhancement of σ-ε properties achieved by SSP up to the first 200 μm, measured from the surface, and allowed the extraction of “layered” elastoplastic stress–strain curves. This is an outstanding achievement for these industrial applications, when considering the total absence of data regarding the elastoplastic properties of decarburized HTT and SSP-affected surface zones of suspension components. A further major benefit of the availability of such curves is their immediate usage within FEM-based SSP simulation models [5] to provide sound understanding of the SSP phenomena occurring in the components’ surface zones and increase the overall SSP simulation accuracy. The exercised work-hardening on the surface layers as an outcome of the severe deformation imposed by the SSP process is herein quantified in a tangible and measurable way, enabling the yield of conclusions with industrial importance and the development of realistic computational models to predict the life span.

## Figures and Tables

**Table 1:** Chemical composition of the 51CrV4 steel grade according to DIN EN 10089.

	C [wt-%]	Si [wt-%]	Mn [wt-%]	P [wt-%]	S [wt-%]	Cr [wt-%]	V [wt-%]
DIN EN 10089	0.47–0.55	≤0.40	0.70–1.10	≤0.025	≤0.025	0.90–1.20	0.10–0.25

**Table 2:** Thickness of the leaf spring at various positions measured from the leaf centre.

**Pos.**	0	90	100	125	150	175	200	250	275	300	350	375	400
**t[mm]**	32.0	32.0	31.72	29.41	26.73	24.92	23.65	21.5	20.38	19.19	16.57	15.61	15.5

## Data Availability

The data presented in this study are available on request from the corresponding author.
